# INNOVATING THE DETECTION OF CARE PRIORITIES IN HEART FAILURE USING LARGE LANGUAGE MODELS

**DOI:** 10.1093/geroni/igae098.4272

**Published:** 2024-12-31

**Authors:** Alaa Albashayreh, Nahid Zeinali, Stephanie White

**Affiliations:** University of Iowa, Iowa City, Iowa, United States; University of Iowa, Iowa City, Iowa, United States; University of Iowa, Iowa City, Iowa, United States

## Abstract

Engaging older adults with advanced chronic conditions, such as heart failure, in discussions about their care priorities is crucial for ensuring treatments align with their preferences, especially at the end of life. Despite the abundance of data in electronic health records (EHRs), documentation of care priorities is often inconsistent and underutilized. This study utilizes natural language processing (NLP) to detect and characterize care priorities in the EHRs of older adults with heart failure, aiming to enhance patient-centered care. We retrained Bio-Clinical-BERT, a Bidirectional Encoder Representations from Transformers (BERT) large language model, using EHR data from a Midwestern U.S. hospital to create Care-BERT, a novel model for predicting care priorities in clinical narratives. We developed a gold-standard corpus of 1,068 notes, focusing on comfort measures only and life-sustaining treatments, with the dataset divided into training (80%) and testing (20%) sets. Care-BERT outperformed BERT-base, Bio-Clinical-BERT, and PubMed-BERT, achieving the highest performance in predicting care priorities (internal validation: F1-score = 0.941, AUC = 0.978; external validation with 200 GPT-based synthetic notes: F1-score = 0.876, AUC = 0.966). Applied to 2,218,251 EHR notes for 7,984 older adults with heart failure (mean age = 76.9 years), Care-BERT revealed that 2.8% had comfort measures only and 17.3% had life-sustaining treatments documented. This study highlights the potential of Transformer-based NLP models like Care-BERT to improve the documentation of care priorities in EHRs and enhance patient-centered care. Future research will explore documentation variations across patient groups using NLP-based labels, providing further insights into care preferences.

